# Cardiac Myosin Binding Protein C Phosphorylation Affects Cross-Bridge Cycle's Elementary Steps in a Site-Specific Manner

**DOI:** 10.1371/journal.pone.0113417

**Published:** 2014-11-24

**Authors:** Li Wang, Sakthivel Sadayappan, Masakata Kawai

**Affiliations:** 1 Department of Anatomy and Cell Biology, College of Medicine, University of Iowa, Iowa City, Iowa, United States of America; 2 School of Nursing, Soochow University, Suzhou, Jiangsu, China; 3 Department of Cell and Molecular Physiology, Health Sciences Division, Loyola University Chicago, Maywood, Illinois, United States of America; Mayo Clinic, United States of America

## Abstract

Based on our recent finding that cardiac myosin binding protein C (cMyBP-C) phosphorylation affects muscle contractility in a site-specific manner, we further studied the force per cross-bridge and the kinetic constants of the elementary steps in the six-state cross-bridge model in cMyBP-C mutated transgenic mice for better understanding of the influence of cMyBP-C phosphorylation on contractile functions. Papillary muscle fibres were dissected from cMyBP-C mutated mice of ADA (Ala273-Asp282-Ala302), DAD (Asp273-Ala282-Asp302), SAS (Ser273-Ala282-Ser302), and t/t (cMyBP-C null) genotypes, and the results were compared to transgenic mice expressing wide-type (WT) cMyBP-C. Sinusoidal analyses were performed with serial concentrations of ATP, phosphate (Pi), and ADP. Both t/t and DAD mutants significantly reduced active tension, force per cross-bridge, apparent rate constant (2π*c*), and the rate constant of cross-bridge detachment. In contrast to the weakened ATP binding and enhanced Pi and ADP release steps in t/t mice, DAD mice showed a decreased ADP release without affecting the ATP binding and the Pi release. ADA showed decreased ADP release, and slightly increased ATP binding and cross-bridge detachment steps, whereas SAS diminished the ATP binding step and accelerated the ADP release step. t/t has the broadest effects with changes in most elementary steps of the cross-bridge cycle, DAD mimics t/t to a large extent, and ADA and SAS predominantly affect the nucleotide binding steps. We conclude that the reduced tension production in DAD and t/t is the result of reduced force per cross-bridge, instead of the less number of strongly attached cross-bridges. We further conclude that cMyBP-C is an allosteric activator of myosin to increase cross-bridge force, and its phosphorylation status modulates the force, which is regulated by variety of protein kinases.

## Introduction

**Figure 1:**
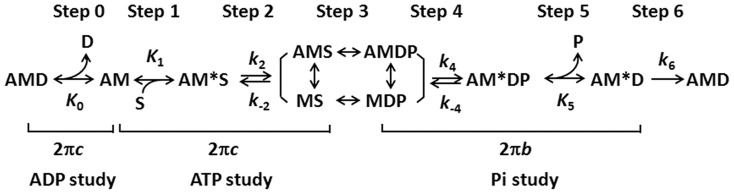
Elementary steps of the cross-bridge cycle in striated muscle fibers. In this scheme, force is generated at step 4 as demonstrated by experiments using rabbit psoas muscle fibers with sinusoidal analyses [22], pressure-release experiments [28], and caged Pi experiments [27]. Step 0: ADP release with the dissociation constant 1/*K*_0_; Step 1: ATP binding with the association constant *K*_1_; Step 2: Cross-bridge detachment and possibly recovery stroke with the forward rate constant *k*_2_, the reversal rate constant *k*_−2_, and the equilibrium constant *K*_2_ = *k*_2_/*k*_−2_; Step 3: ATP cleavage followed by a formation of weakly attached/detached cross-bridges; Step 4: Pi isomerization and force generation with the forward rate constant *k*_4_, the reversal rate constant *k*_−4_, and the equilibrium constant *K*_4_ = *k*_4_/*k*_−4_; Step 5: Pi release with the dissociation constant 1/*K*_5_; Step 6: ADP isomerization and work performance (filament sliding). A = Actin, M = Myosin, D = ADP, S = ATP, and P = phosphate.

Muscle contraction is achieved by the cyclic interaction between myosin cross-bridges of the thick filament and actin on the thin filament, during which the actin-myosin-ATP complex undergoes several different states to achieve the transduction of chemical energy stored in ATP to mechanical work. This is called the cross-bridge cycle, and each step within the cycle is called an elementary step. A complete cross-bridge model which include six elementary steps is described in Fig. 1 (Scheme 8 in [1]). From experiments that observe the effects of ATP, phosphate (Pi), and ADP on tension transients, the kinetic constants of the elementary steps can be characterized [2].

Myosin binding protein C (MyBP-C), as an integral part of the thick filament [3], is implicated in both regulatory and structural functions in striated muscles [4]. Its cardiac isoform (cMyBP-C) can be phosphorylated at multiple sites located in the M domain between domains C1 and C2 [5], [6]. The phosphorylation status of cMyBP-C is associated with various pathological conditions in the heart, and plays a critical role in regulating force generation by modulating the thick-to-thin filament interaction [6]–[9]. Three hierarchically accessible and functionally unequal phosphorylation sites, Ser(S)273, 282 and 302, have been identified in murine and are homologous to humans [10]–[13]. The pattern of phosphorylation at the three sites is not random, and has shown mutual interactions which can be positive or negative [14]. Among the three sites, S282 is phosphorylated first before phosphorylation takes place at S273 and S302 [10], hence S282 is thought to play a critical role in the phosphorylation of cMyBP-C [13].

The histological characteristics and cardiac function at the whole organ level were studied in cMyBP-C transgenic mice of ADA (Ala273-Asp282-Ala302), DAD (Asp273-Ala282-Asp302), SAS (Ser273-Ala282-Ser302), and wild-type (WT: Ser273-Ser282-Ser302) genotypes (Ala: phospho-ablation; Asp: phosphomimetic), which were created on the cMyBP-C knockout (t/t) background [13]. The Asp or Glu mutant mimics the phosphorylated state of Ser; and alanine, an amino acid that is structurally similar to Ser, is the common replacement for phospho-ablation [15]. Although the t/t phenotype can be completely rescued by WT, it can only be partially rescued by ADA, DAD and SAS [13]. Among them, mouse hearts from DAD show significant hypertrophy, fibrosis, and myocyte disarray, mimicking the t/t phenotype [13]. A recent study found that DAA and AAD mice exhibited left ventricular dilation, interstitial fibrosis, irregular cardiac rhythm, and sudden cardiac death, suggesting that the normal phosphorylation status of the three Ser residues is essential for cardiac function, and that chronic phosphorylation at one site, with the inability to phosphorylate the other sites, can be detrimental to the maintenance of normal cardiac structure and function [16].

With the importance of cMyBP-C phosphorylation in contraction, regulation, and the hierarchically functional S273, S282 and S302 sites in mind, we have recently found that phospho-aberrant (nonphosphorylatable) Ala282 combined with phosphomimetic residues Asp273 and/or Asp302 (in DAD) is detrimental to cardiomyocytes as evidenced by the lower active isometric tension and altered cross-bridge kinetics with decreased 2π*c* (fast rate constant) and increased 2π*b* (medium rate constant). On the other hand, a single change of residue 282 to phospho-aberrant Ala (SAS), or to phosphomimetic Asp together with changes of residues 273 and 302 to nonphosphorylatable Ala (ADA), caused a minute change in fibre mechanics [17]. Based on these findings, we aimed at studying further the site-specific effect of cMyBP-C phosphorylation on cross-bridge force and elementary steps of the cross-bridge cycle (Fig. 1). For this purpose, different concentrations of ATP (0.05–10 mM), Pi (0–30 mM), and ADP (0–3 mM) were applied to skinned cardiac papillary and trabecular muscle fibres. During Ca^2+^ activation, small amplitude sinusoidal length changes were applied to the fibres and the tension transients were recorded, through which the kinetic constants were deduced, and the elementary steps as well as force per cross-bridge were characterized. We found that the reduced tension production in DAD and t/t is the result of reduced force per cross-bridge, instead of the less number of strongly attached cross-bridges, emphasizing the significance of cMyBP-C in allosteric activation of cross-bridge force.

## Materials and Methods

### 1. Animals

All the animal experiment protocols were approved by the Institutional Animal Care and Use Committees at Loyola University Chicago, conforms the contents of *Guide for the Use and Care of Laboratory Animals* published by the National Institutes of Health. The transgenic mouse models used in the present study were in the t/t background lacking endogenous cMyBP-C, and they have been reported in our previous investigations [17]. t/t is a hypertrophic cardiomyopathy mutant mouse with C-terminal modifications that causes an absence of cMyBP-C in sarcomeres [18]. WT mice were crossed on the t/t background, and used as the control. Totally 16 mice of WT, t/t, ADA, DAD, and SAS models were tested. The details of the mouse models, the information on the number of mice used, their age and gender, can be found in Wang et al [17].

### 2. Muscle fibre preparations

Skinned mouse cardiac papillary and trabecular muscle fibres (strips) were prepared from both ventricles of the heart that was removed after the mice were euthanized through intraperitoneal injection of Na pentobarbital at 50 mg/kg. All efforts were made to minimize potential pain, suffering or distress. The method is same as previously described [17].

### 3. Experimental procedure

Experimental procedure is similar to the one in our previous study [19]. Fibres were dissected and mounted on the experimental apparatus by attaching their ends to two stainless-steel hooks with a small amount of nail polish. One hook was connected to a length driver to change the fibre length, and the other to a tension transducer to detect isometric tension and its transients. After mounting, fibres were soaked in relaxing solution for a few minutes and thereafter further skinning for 20 min in the relaxing solution to which 1% Triton X-100 was added. Before applying activating solutions for mechanical measurements, fibres were washed with the relaxing solution and their length was adjusted to remove the slack. This achieved sarcomere length of 2.1–2.2 µm, as measured by laser diffraction [20]. ATP, Pi, and/or ADP studies were performed on the fibres to determine the kinetic constants of the elementary steps based on the six-state cross-bridge model (Fig. 1). In these studies, activating solutions contain a series of concentration of ATP (*S*: 0.05–10 mM), Pi (*P*: 0–30 mM), or ADP (*D*: 0–3 mM). The rigor stiffness was tested in the end. The solution compositions are listed in Table 1. All experiments were performed at 20°C and a circulating water bath system was used to maintain the constant temperature.

**Table 1:** Solution compositions.

Ingredient (mM)	Relaxing	ATP study		Pi study		ADP study			Rigor solution
		0S	10S	0P	30P	00D	0D	3D	
K_2_CaEGTA	-	6	6	6	6	6	6	6	-
K_2_H_2_EGTA	6	-	-	-	-	-	-	-	-
Na_2_H_2_ATP	7	0	12.12	6.12	6.058	2.9	2.93	2.86	-
NaADP	-	-	-	-	-	-	0	11.84	-
A_2_P_5_	-	-	-	-	-	-	0.1	0.1	-
Na_2_CP	-	15	15	15	15	15	-	-	-
HK_2_PO_4_+H_2_KPO_4_ 1	8	8	8	0	30	8	8	8	8
MgAc_2_	2	1.69	11.54	6.68	6.44	2.83	2.75	5.67	-
NaAc	41	25	0.77	12.77	12.89	9.2	39.1	27.4	55
KAc	70.5	74	33.23	72.08	2.54	77	91.8	50.7	122
KCl	-	12	12	12	12	-	-	-	-
KOH	19	5.4	29.37	15.82	21.26	5.8	5.86	25.8	5
MOPS	10	10	10	10	10	10	10	10	10
NaN_3_	-	-	-	-	-	10	10	10	-
CK (U/ml)	-	80	80	80	80	80	-	-	-

1 Equimolar mixture.

Ac = acetate, CP = creatine phosphate, Pi = phosphate, CK = creatine kinase. Ionic strength of all solutions is 200 mM. pCa of activating solutions is 4.34–4.65, and that of relaxing solution is>9. For activating solutions, [Mg^2+^] 1 mM; [MgATP^2−^] in the *η*S solution is *η* mM, in *η*D solution is 2 mM, and in *η*P solution is 5 mM; [Na^+^] 55 mM, and pH is adjusted to 7.00±0.02 by KOH. Solution 0S and 10S were mixed the by ratio (10-*η*):*η* to make the *η*S solution used in the ATP study. Solution 0P and 30P were mixed the by ratio (30-*η*):*η* to make the *η*P solution used in the Pi study. Similarly, solution 0D and 3D were mixed the by ratio (3-*η*):*η* to make the *η*D solution used in the ADP study.

### 4. Sinusoidal analysis

Sinusoidal analysis method has been described in our previous publications [17], [19]. In the current study, the mouse cardiac muscle fibres are studied at 20°C. The frequency (*f*) response function *Y*(*f*) (complex modulus) of the tension change to the stress change is resolved into the sum of two exponential processes B and C. 2π*b* is the apparent rate constant of process B, called delayed tension; and 2π*c* is the apparent rate constant of processs C, called fast tension recovery. They are associated with the active interaction of myosin cross-bridges with actin which leads to chemomechanical energy transduction [21]. The parameters of exponential processes were studied in the present study as functions of [ATP], [Pi], and [ADP] to deduce the kinetic constants of elementary steps of the cross-bridge cycle and as reported [22]. *Y*(∞) is generally called “stiffness” in this report. A detailed description of the sinusoidal analysis method was published previously [21].

### 5. ATP study

In the ATP study, through applying different concentrations of ATP (0.05, 0.1, 0.2, 0.5, 1, 2, 5, and 10 mM) to the skinned fibres and recording tension and its transients during each activation at pCa 4.34–4.65, the exponential process C was studied aiming at observing the kinetic constants of the ATP binding step 1 (*K*_1_), and the subsequent rapid cross-bridge detachment step 2 (*k*_2_ and *k*_−2_) (Fig. 1). The apparent rate constant 2π*c* was deduced from *Y*(*f*) during activation at each ATP concentration. The relationship between 2π*c* and [ATP] were fitted to Eq. 1 which was developed based on the cross-bridge model in Fig. 1, where *S* represents [ATP], *D* represents [ADP] and *D*_0_ represents the contaminating [ADP] in solutions and fibres. Because of the presence of creatine phosphate (CP) and creatine kinase (CK) (Table 1) in the ATP study, *K*_0_ (*D*+*D*_0_) in Eq. 1 was assumed to be 0 as the first approximation.
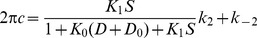
(1)(Eq. 1 is based on Eq. 16 of [22].)

### 6. Pi study

The effect of Pi on the exponential processes B and C and isometric tension were studied through applying 0, 2, 4, 8, 16, and 30 mM Pi to the fibres, with the purpose to investigate the effects of cMyBP-C mutants on the force generation step 4 and the Pi release step 5 (Fig. 1). The sum of the two apparent rate constants (2π*b*+2π*c*) was deduced from the Pi study, and the relationships between [Pi] and (2π*b*+2π*c*), and [Pi] and tension were fitted to Eqs. 2 and 3, respectively. These equations were derived based on the cross-bridge model in (Fig. 1) [22]:
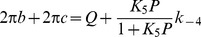
(2)(Eq. 2 comes from Eq. 14 of [22].)
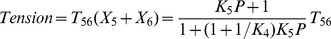
(3)(Eq. 3 is derived from Eq. 5 of [23] by setting *T*_5_ = *T*_6_ = *T*_56_.)

where *P* represents [Pi], *X*_5_ and *X*_6_ represent the probabilities of cross-bridges in the AM*DP and AM*D states, respectively, and *T*_56_ is the tension supported by these states, i.e., the tension when all the cross-bridges are distributed either at *X*_5_ or *X*_6_, and it is proportional to force per cross-bridge. *k*_−4_ (rate constant of the reverse force generation step) and *K*_5_ (Pi association constant) were first determined by fitting the rate constant data to Eq. 2. Then, based on this *K*_5_, the tension data were fitted to Eq. 3 to determine *K*_4_ (equilibrium constant of force generation step) and *T*_56_. *k*_4_ is calculated as *k*_4_ = *K*_4_ *k*_−4_ in the usual way.

### 7. ADP study

To characterize the association constant (*K*_0_) of ADP to cross-bridges, the effect of ADP was studied through applying 0–3 mM ADP to muscle fibres, in which [ATP] was fixed to 2 mM and [Pi] to 8 mM (Table 1). By fitting the relationship between the rate constant 2π*c* and [ADP] to Eq. 1, *K*_0_ and *D*_0_ were deduced. Here we used *K*_1_, *k*_2_, and *k*_−2_ derived from the ATP study, and *S* = 2 mM (experimental condition). During the ADP study, 00*D* solution, which contained CP and CK but no A_2_P_5_ nor ADP, was first applied to the fibres, followed by the 0*D* solution, which did not contain CP, CK, or ADP, but contained A_2_P_5_ (adenyl kinase inhibitor). The 1*D*, 2*D* and 3*D* solutions were applied sequentially thereafter. These solutions enable to find out the contaminating ADP concentration (*D*_0_) in the 0D–3D solutions by extrapolation.

### 8. Cross-bridge distribution

Based on Eqs. 10–13 of [24], the cross-bridge distribution at AMDP (X_34_), AM*DP (X_5_) and AM*D (X_6_) states in (Fig. 1) was calculated under the activating conditions.

### 9. Rigor stiffness

The rigor state was induced at the end of experiments by applying the rigor solution twice, and rigor stiffness was measured at 100 Hz.

### 10. Myofilament proteins' phosphorylation levels

Pro-Q Diamond dye provides the capability of quantifying the protein phosphorylation levels on a proteome-wide scale through the fluorescence detection of phosphoserine-, phosphothreonine-, and phosphotyrosine-containing proteins directly in sodium dodecyl sulfate (SDS)-polyacrylamide gels and two-dimensional (2-D) gels [25]. The fluorescence signal intensity correlates with the number of phosphorylated residues on the protein [25]. The pseudophosphorylation with Glu or Asp is undetected by this method owing to the absence of phosphate. The phosphorylation levels of cMyBP-C, cardiac troponin T (cTnT), cardiac troponin I (cTnI), tropomyosin (Tm), and myosin regulatory light chain (RLC) were analyzed by Pro-Q Diamond-stained gels and normalized against SYPRO Ruby-stained total protein levels. The procedure of Pro-Q Diamond and SYPRO Ruby staining have been previously described [26]. The protein phosphorylation levels in each mouse model were expressed in a value normalized to the average level of WT. All proteins were quantified in five mouse models, including WT, t/t, ADA, DAD, and SAS, with 2–3 mice in each model.

### 11. Statistics

All the data were expressed as mean ± standard error (SE). One-way analysis of variance (ANOVA) was applied to determine the significance of the difference among different groups of muscle preparations, with post-hoc range tests (least significant difference, LSD) using pair wise multiple comparisons. A significant difference was defined as *0.01<*P*≤0.05, and a highly significant difference as **P≤0.01. A slight effect was defined as ^(^*^)^ 0.05<*P*≤0.1.

## Results

### 1. The ATP binding and cross-bridge detachment steps (steps 1 and 2 in Fig. 1)

**Figure 2:**
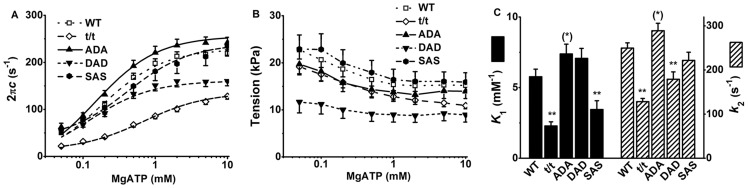
Results of the ATP study and parameters deduced from it. (A) Apparent rate constant 2π*c* versus [ATP] plot, (B) active tension versus [ATP] plot, and (C) the ATP association constant (*K*_1_) and the forward rate constant of the cross-bridge detachment step (*k*_2_). *n* = 20, 34, 21, 14, and 14 for WT, t/t, ADA, DAD, and SAS mice, respectively. In (A), the continuous lines are the best fit curves to Eq. 1. 2πc was significantly smaller in t/t than in WT from 0.05S to 10S. DAD showed significantly smaller 2πc in the high concentration range of ATP (0.5S–10S). In (B), tension production in DAD mice was significantly lower than that in WT, whereas the lower tension in t/t than WT did not reach the significant level. In (C), **P≤0.01, and ^(^*^)^ 0.05<*P*≤0.1 when compared with WT.

During Ca^2+^ activation at varied ATP concentration, isometric tension was measured, sinusoidal analysis was performed to obtain the complex modulus data *Y*(*f*), and the rate constant of the exponential process C (2π*c*) was derived from *Y*(*f*) as described previously [21]. These are plotted in Fig. 2A in the semi log scale. 2π*c* increased when [ATP] was increased from 0.05 mM to 1 mM, and saturated by 10 mM (Fig. 2A). The continuous curves in Fig. 2A represent the best fit curves to Eqs. 1. This hyperbolic (sigmoid in the semi log scale) relationship between 2π*c* and [ATP] is a common character of skeletal and cardiac muscle fibres [19], [23]. The t/t and DAD mice showed significantly decreased 2π*c* compared to WT. Isometric tension decreased with the increase of [ATP] as shown in (Fig. 2B). DAD had the lowest tension, followed by t/t.

Through fitting the relationship between 2π*c* and [ATP] to Eq. 1, the kinetic constants surrounding step 1 (*K*_1_) and step 2 (*k*_2_) were deduced and plotted in (Fig. 2C). Compared with WT, t/t and SAS mice showed significantly decreased ATP association constant (*K*_1_), t/t and DAD showed significantly decreased *k*_2_ (forward rate constant of the cross-bridge detachment step 2), whereas ADA group showed somewhat opposite effects as evidenced by the slightly increased *K*_1_ (*P* = 0.1) and *k*_2_ (*P* = 0.09) (Fig. 2C). Because 2π*c* extrapolated near to the origin in the linear plot of (Fig. 2A), equilibrium constant of the cross-bridge detachment step (*K*_2_) was not calculated. This indicates that *k*_−2_ is very small, hence *K*_2_ is very large.

### 2. The force generation and Pi release steps (steps 4 and 5 in Fig. 1)

**Figure 3:**
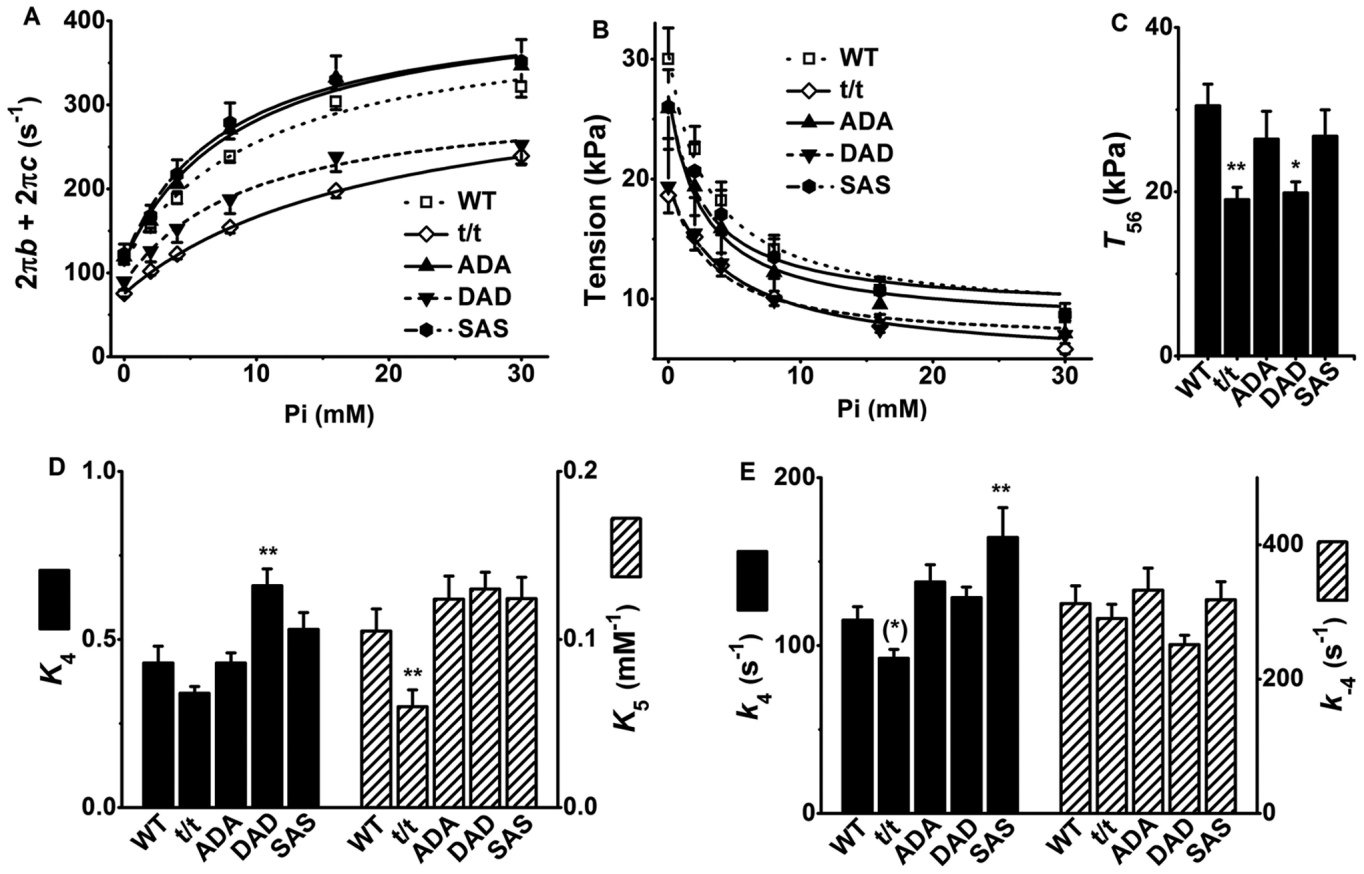
Results of the Pi study and parameters deduced from it. (A) Sum of the apparent rate constants (2π*b*+2π*c*) versus [Pi] plot, (B) tension versus [Pi] plot, (C) force per cross-bridge (*T*_56_), (D) equilibrium constants of force generation (*K*_4_) and Pi association (*K*_5_), and (E) the forward (*k*_4_) and reverse (*k*_−4_) rate constants of the force generation step. *n* = 17, 28, 13, 9, and 12 for WT, t/t, ADA, DAD and SAS mice, respectively. The continuous lines in (A) are the best fit curves to Eq. 2, and the continuous lines in (B) are the best fit curves to Eq. 3. **P≤0.01, *0.01<*P*≤0.05, and ^(^*^)^ 0.05<*P*≤0.1 when compared with WT.

During Ca^2+^ activation at varied Pi concentration, isometric tension was measured, sinusoidal analysis was performed to obtain Y(*f*), and the rate constant (2π*b*) of process B and the rate constant (2π*c*) of process C were derived. These are plotted in (Figs. 3A) (2π*b*+2π*c*) and B (tension). The continuous curves represent the best fit curves to Eqs. 2 and 3. Similar to the ATP study, both t/t and DAD mutants produced significantly smaller apparent rate constants (2π*b*+2π*c*) at 0–30P with the lowest values in t/t mice. The tension at each [Pi] was significantly less in both DAD and t/t mice (*P*≤0.05 in t/t and DAD except at 30P in DAD).

Through the sequential fitting the relationship between [Pi] and (2π*b*+2π*c*) to Eq. 2, and relationship between [Pi] and tension to Eq. 3, the kinetic constants surrounding step 4 (*k*_4_ and *k*_−4_) and step 5 (*K*_5_) as well as the force per cross-bridge (*T*_56_) were deduced (Fig. 3C–E). Here *K*_5_ is defined as the Pi association constant, and its dissociation constant is 1/*K*_5_. Despite the slightly decreased *k*_4_ in t/t and significantly increased *k*_4_ in SAS (Fig. 3E), there was no significant change found in *K*_4_ in t/t and SAS (Fig. 3D). In contrast, in DAD, the slightly elevated *k*_4_ and the slightly reduced *k*_−4_ (Fig. 3E) resulted in a significant increase in *K*_4_ (Fig. 3D). *K*_5_ was significantly decreased in t/t (Fig. 3D), indicating the Pi release was enhanced in the t/t mouse model. Both t/t and DAD mice displayed significantly smaller *T*_56_, indicating the smaller force production per cross-bridge (Fig. 3C). Previously, tension with the standard activation (8 mM Pi) in t/t was less than that of WT, but their difference was insignificant (Fig. 3 of [17]). With the increased number of the Pi points between 0 mM and 30 mM, and fitting all the data points available (n = 168), we now conclude that force/cross-bridge (*T*_56_) is less in t/t than in WT, and this difference is significant.

### 3. The ADP release step (step 0 in Fig. 1)

**Figure 4:**
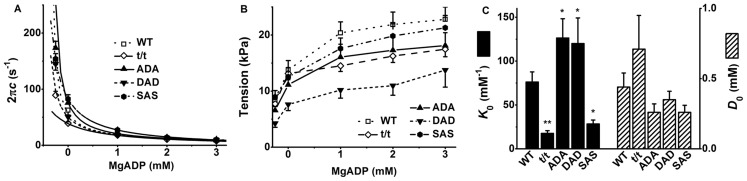
Results of the ADP study and parameters deduced from it. (A) 2π*c* versus [ADP] plot, (B) tension versus [ADP] plot, and (C) the ADP association constant (*K*_0_) and ADP contamination (*D*_0_). *n* = 12, 19, 12, 10, and 8 for WT, t/t, ADA, DAD, and SAS mice, respectively. The continuous lines in (A) are the best fit curves to Eq. 1. **P≤0.01 and *0.01<*P*≤0.05 when compared with WT.

In contrast to the findings in the ATP study, the rate constant 2π*c* decreased and the tension increased as [ADP] increased in all groups of muscle fibres in the ADP study (Fig. 4A–B). t/t exhibited significantly smaller 2π*c* than that of WT at 00D (*P*≤0.01), 0*D* (*P*≤0.05) and 3D (*P*≤0.05), whereas DAD showed unaltered 2π*c*. On the other hand, SAS exhibited a larger value of 2π*c* than WT at 1D (*P*≤0.01). The active tension was significantly less in DAD (*P*≤0.01 at all ADP concentration) and t/t (*P*≤0.05 at 1D and 2D, *P* = 0.056 at 3D) than that of WT mice (Fig. 4B). t/t and SAS displayed significantly smaller *K*_0_, whereas ADA and DAD exhibited larger *K*_0_ than that of WT (Fig. 4C). Here *K*_0_ is defined as the ADP association constant, and its dissociation constant is 1/*K*_0_.

In the 00*D* solution, the rate constant 2π*c* was larger (Fig. 4A), and tension was less (Fig. 4B) than those in the 0D solution, demonstrating that the contaminating [ADP] in the 0D–3D solutions is significant. The contaminating ADP concentration (*D*_0_) in the 0D–3D solutions, which resulted from ATP hydrolysis by muscle fibres and from ADP contamination present in ATP, was evaluated by extrapolating the 2π*c* data versus [ADP] (Fig. 4A, continuous curves) to the value of 2π*c* obtained in the 00D solution. The corresponding abscissa value is –*D*_0_. We found that *D*_0_ (in mM) was 0.44±0.10 in WT (*n* = 12), 0.71±0.24 in t/t (*n* = 19), 0.26±0.06 in ADA (*n* = 12), 0.35±0.06 in DAD (*n* = 10), and 0.26±0.05 in SAS (*n* = 8). Thus, 0.3–0.7 mM extra ADP was present in the 0D–3D solutions which do not have the ATP regenerating system. This extra ADP was taken into consideration when calculating *K*_0_, as shown in Eq. 1.

### 4. Cross-bridge distributions

**Figure 5:**
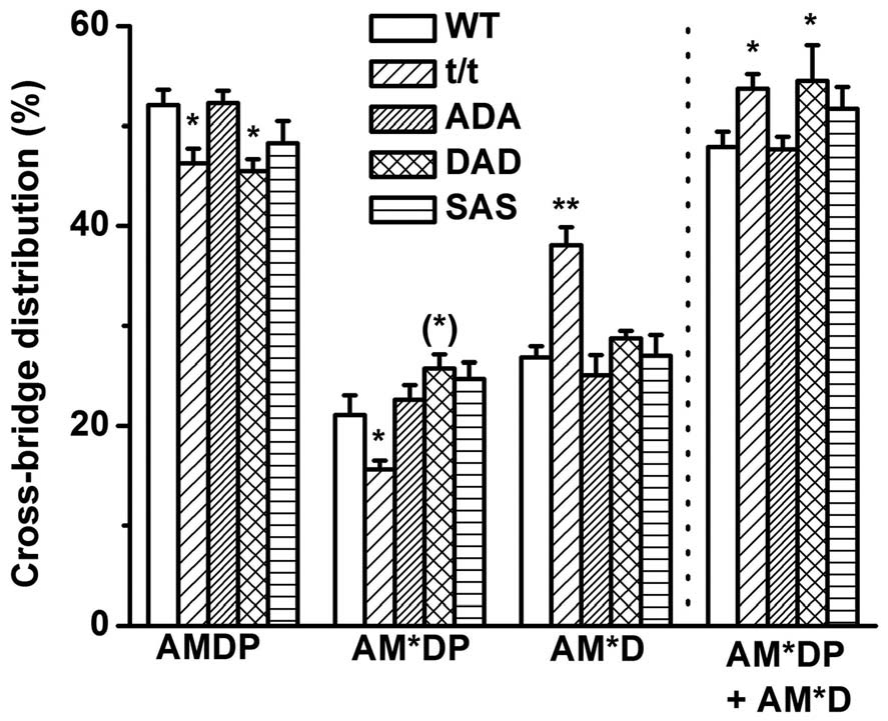
The cross-bridge distribution at the standard activating condition (*S* = 5 mM and *P* = 8 mM) among the AMDP, AM*DP, and AM*D states. **P≤0.01, *0.01<*P*≤0.05, and ^(^*^)^ 0.05<*P*≤0.1 when compared with WT. AM*DP+AM*D indicate the total strongly attached cross-bridges.

To determine the number of force generating cross-bridges, the cross-bridge distribution was calculated among the AMDP, AM*DP, and AM*D states (Fig. 5). AMDP isomerizes to form AM*DP state and force is generated during this isomerization step [22], [23], [27], [28], hence, AM*DP is a strongly attached state. The force generation (step 4) is followed by Pi release (step 5) to form the AM*D state, which maintains the same amount of force as the AM*DP state. Because of this, the distributions at AM*DP and AM*D are summated together and included in Fig. 5 to represent the number of strongly attached cross-bridges. t/t and DAD had more cross-bridge distributions than WT at the strongly attached state. ADA and SAS had no significant changes from WT in any of the states we observed (Fig. 5).

### 5. Rigor stiffness

**Figure 6:**
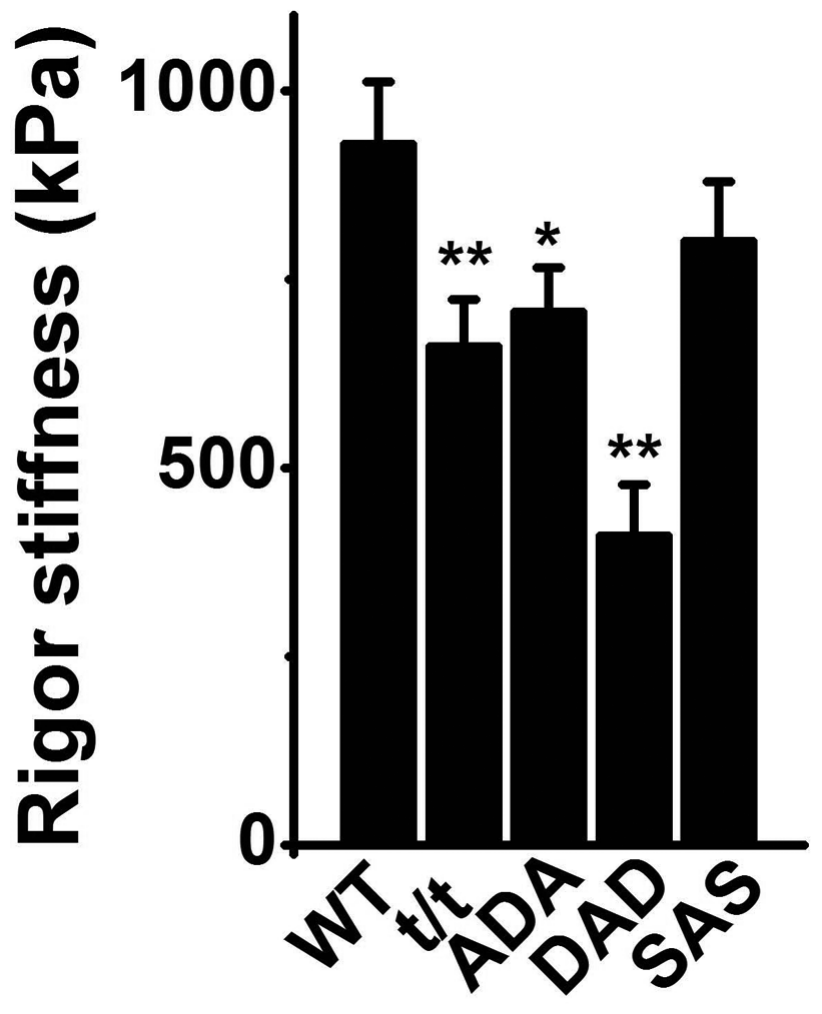
Rigor stiffness measured at 100 Hz. **P≤0.01 and *0.01<*P*≤0.05 when compared with WT.

To determine the strength of the actomyosin interaction and its associated change with the cMyBP-C mutants, rigor stiffness was measured and plotted in Fig. 6. The rigor stiffness is highly significantly decreased in t/t and DAD (P≤0.01), and significantly decreased in ADA (P≤0.05) compared to WT (Fig. 6).

### 6. Myofilament protein phosphorylation levels

**Figure 7:**
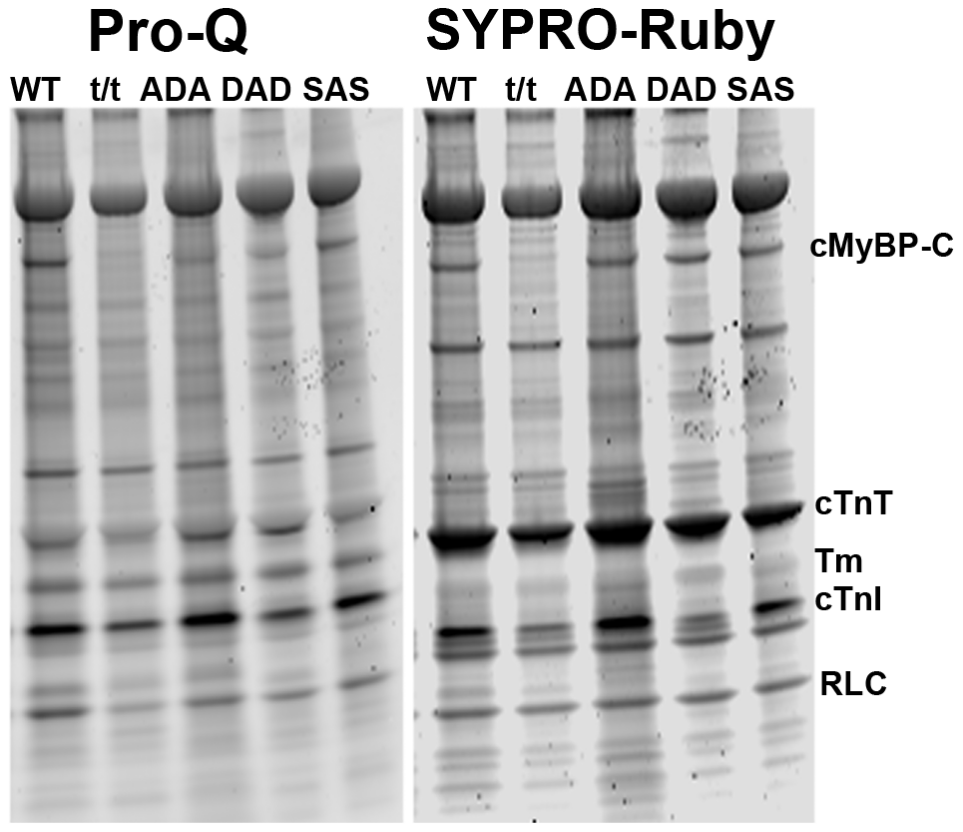
The representative gel of Pro-Q Diamond staining (*left*, phosphorylation) and SYPRO-Ruby staining (*right*, total protein).

**Figure 8:**
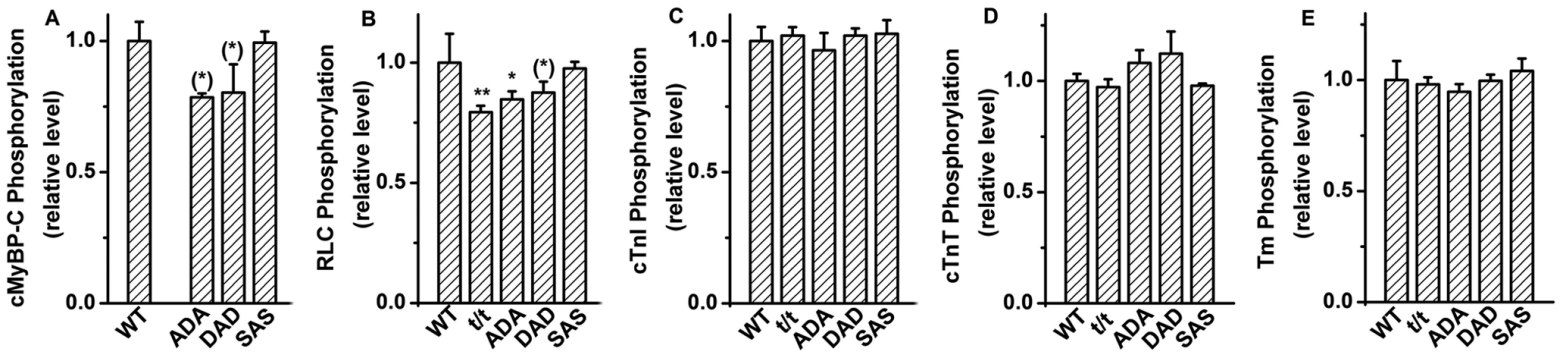
Protein phosphorylation level. (A) cMyBP-C, (B) RLC, (C) cTnI, (D) cTnT, and (E) Tm. **P≤0.01, *0.01<*P*≤0.05, and ^(^*^)^ 0.05<*P*≤0.1 when compared with WT.

To find out the influence of cMyBP-C mutations on phosphorylation of other myofilament proteins as well as cMyBP-C itself, cardiac muscle tissues were subjected to Pro-Q Diamond staining first, followed by SYPRO-Ruby staining. The representative gel from Pro-Q staining for phosphorylated protein and SYPRO-Ruby staining for total protein is shown in Fig. 7. The band densities were calculated using Image-J software, and the relative phosphorylation levels of cMyBP-C, RLC, cTnI, cTnT, and Tm proteins are plotted in Fig. 8. The band of cMyBP-C was missing in t/t mouse from either Pro-Q Diamond or SYPRO-Ruby staining due to the cMyBP-C null genotype (Figs. 7 and 8A). The phosphorylation level of cMyBP-C was slightly decreased in ADA and DAD, but not changed in SAS compared to WT (Fig. 8A). The phosphorylation level of RLC was significantly decreased in t/t and ADA, slightly decreased in DAD, but not changed in SAS compared to WT (Fig. 8B). The phosphorylation levels of cTnI, cTnT and Tm (Fig. 8C–D) were not significantly different among all transgenic mouse models studied. In our study, muscle samples from WT and mutated mice were processed at the same time with the same procedures for comparability. We have shown that the phosphorylation levels do not change with time in skinned fibers, as we have demonstrated over the 8 month period (Fig. 3 of [29]).

## Discussion

The present project aimed at finding the function of cMyBP-C phosphorylation in cardiac contractile mechanisms through characterizing the elementary steps of the cross-bridge cycle in cMyBP-C's phosphorylation site-mutated mice. Our study demonstrates that mutations of cMyBP-C's different phosphorylation site(s) differentially affect cross-bridge kinetics. Papillary muscle fibres from t/t mice showed the most broad effect with decreased force per cross-bridge (*T*_56_, Fig. 3C), rigor stiffness (Fig. 6), cross-bridge detachment rate (*k*_2_) (Fig. 2C), the ATP (*K*_1_) (Fig. 2C), Pi (*K*_5_, Fig. 3D), and ADP (*K*_0_, Fig. 4C) association constants. DAD mice, carrying phospho-ablated S282 and phospho-mimetic S273 and S302 in cMyBP-C, had similar effects with t/t in decreased force per cross-bridge (*T*_56_, Fig. 3C), decreased rigor stiffness (Fig. 6), decreased cross-bridge detachment rate (*k*_2_, Fig. 2C), but had opposite changes from t/t in increased equilibrium constant of the force generation step (*K*_4_, Fig. 3D), the increased ADP association step (*K*_0_, Fig. 4C) without influencing the ATP binding (*K*_1_, Fig. 2C) or Pi release step (*K*_5_, Fig. 3D). ADA (phospho-mimetic S282 with phospho-ablated S273 and S302) mice showed decreased rigor stiffness (Fig. 6), increased ADP association (Fig. 4C), and the slightly increased ATP association (*K*_1_) and the slightly increased cross-bridge detachment rate (*k*_2_) (Fig. 2C). SAS (phospho-ablated S282) mice showed the decreased ATP (Fig. 2C) and ADP (Fig. 4C) association constants.

It has been observed that homozygous t/t mutant mice exhibited left ventricular dilation and reduced contractile function at birth, and progressed to a dilated cardiomyopathy with the reduced ejection fraction and maximal left ventricular end-systolic elastance, despite a preserved maximal rate of pressure rise; the myocardial hypertrophy also increased as animals matured [30]. All ADA, DAD, and SAS mice have significantly increased interventricular septal thickness, increased left ventricular end-diastolic and end systolic dimensions, and variable extent of reduced fractional shortening [13]. Cardiac hypertrophy with fibrosis and myocyte disarray was observed in t/t and DAD mice [13]. In the present study, we found that t/t has the most profound effects and affected every elementary step involved in the cross-bridge cycle, hence the contractility represented by active tension, stiffness, and force per cross-bridge is significantly affected. In contrast, ADA, DAD, and SAS affected only limited steps in the cross-bridge cycle. These observations are consistent with the previous finding that the ADA, DAD and SAS partially rescued t/t phenotype [13]. However, the similar changes between t/t and DAD in tension, the apparent rate constant 2π*c* and the sum (2π*b*+2π*c*), and the rate constant of the cross-bridge detachment step (*k*_2_) suggest that the DAD mice mimic the t/t mice in many mechanical profiles. The significantly smaller *T*_56_ (force produced/supported by each cross-bridge, Fig. 3C) is the reason for the decreased tension production in t/t and DAD, since the number of strongly attached cross-bridges at [AM*DP] and [AM*D] is larger in t/t and DAD mice than those in WT mice (Fig. 5). The similarly decreased force per cross-bridge in DAD and t/t mutants reinforces our previous conclusion that the protein kinase C (PKC) mediated S273 and S302 phosphorylation adversely affects the cross-bridge cycle and cardiac contraction [13].

Significantly decreased rate constant 2π*c* in the ATP and ADP studies (Figs. 2A and 4A, respectively) and decreased 2π*b*+2π*c* in the Pi study (Fig. 3A) were found in t/t in the present study. DAD mice showed similar changes (smaller 2π*c* and 2π*b*+2π*c*; Figs. 2A, 3A, and 4A). These observations do not mean, however, that 2π*b* decreases in t/t and DAD mice: because 2π*b* is much smaller than 2π*c* (2π*c* is 2–4 times of 2π*b*), their sum (in the Pi study) is mostly governed by 2π*c*. In fact, in the previous study, we found an increased rate constant 2π*b* and a decreased rate constant 2π*c* in DAD with the standard activation (see Fig. 4A of [17]), which made us to hypothesize that the cross-bridge detachment step(s) was decelerated, and/or the cross-bridge attachment step(s) was accelerated to result in a larger number of strongly attached cross-bridges with PKC sites (S273 and S302) phosphorylation. Our current finding, that *k*_2_ (rate constant of cross-bridge detachment) is significantly less and *K*_4_ (equilibrium constant of cross-bridge attachment/force generation step) is significantly more in DAD than WT (Figs. 2C and 3C), are in accord with our earlier hypothesis, which is also demonstrated in Fig. 5 that the number of strongly attached cross-bridges are more in DAD than in WT.

DAD shows some different effects from t/t: all ligand association constants (*K*_0_, *K*_1_, and *K*_5_) are larger in DAD than those of t/t (Figs. 2C, 3D, and 4C). These effects indicate that the presence of cMyBP-C and its phosphorylation status significantly affect the nucleotide and Pi binding sites of myosin, indicating that there is a direct contact between cMyBP-C and the myosin head, or the signal is transmitted from the cMyBP-C binding site through the lever arm to the myosin head. The significantly larger *K*_4_ in DAD than in t/t (Fig. 3D) contributes to the transition of AMDP (weak) to AM*DP (strong) and causes more cross-bridges at the AM*DP state than in WT, and as shown in Fig. 5. The increased *K*_5_ in DAD compared to t/t suggests that the Pi release decreases, causing less cross-bridges to transform from AM*DP to AM*D, a fact that can be seen as an inversion of the cross-bridge distributions in the AM*D state (Fig. 5). In all, we conclude that a large (∼50%) tension and stiffness decrease in DAD is primarily due to a decrease in force per cross-ridge (Fig. 3), and the small increase in the number of strongly attached cross-bridges cannot compensate for this decrease (Fig. 5).

While t/t and DAD have large effects on tension and cross-bridge kinetics, the effects induced by ADA and SAS are small. In ADA, S273 and S302 are phospho-ablated, and in SAS the phosphorylation of S273 and S302 are strongly inhibited due to the phospho-ablated S282. S282 phosphorylation has been shown to play a leading role in the phosphorylation of other sites [10], [13]. In ADA, with phospho-mimetic S282 and phospho-ablated S273 and S302, the significantly increased association constant for ADP (*K*_0_, Fig. 4C) causes a reduced ADP release resulting in a slower sarcomere shortening. This is because the shortening velocity is controlled by the rate at which ADP can escape from cross-bridges after completion of the power stroke [31]. There is a strong correlation between the maximum ATP hydrolysis rate (V_max_) and the rate of ADP release from actomyosin [32]. These observations may account for the reduced systolic function found in ADA mice [13]. In SAS with phospho-ablated S282, the result is opposite to ADA, as shown by the decreased *K*_0_ and *K*_1_ (Fig. 4C and 2C). No apparent abnormalities: fibrosis, calcification, or disarray, were found in the ADA or SAS mouse phenotype [13]. Therefore, the results in muscle mechanics in the present study and the histological results [13] are consistent. ADA and SAS produced small effects in cross-bridge kinetics with the only effects in *K*_0_ and *K*_1_; the increase in *k*_4_ in SAS did not result in a significant change in *K*_4_. The fact that ADA and SAS affect the ATP binding and ADP release steps, and DAD affects multiple steps demonstrates that a different mutant affects different elementary steps in the cross-bridge cycle. We have further found that t/t and DAD are more harmful mutations to cause detrimental effects than ADA and SAS mutations.

The fact that only central 9/17 (53%, c-zone) of the thick filament accompanies cMyBP-C, and the remaining 8/17 (47%, b-zone for cMyBP-C “bare” zone) does not accompany cMyBP-C [33] needs a consideration. Because cross-bridges in the two zones are mechanically in parallel, their total force (*F*, measured force) is the weighted sum of individual forces (*F*_c_ and *F*_b_, respectively, which are force per unit length of the thick filament):
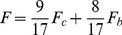
(4)Where *F*_c_ is force if all the cross-bridges are accompanied with cMyBP-C, and *F*_b_ is force if none of the cross-bridges are accompanied with cMyBP-C. *F*_c_ and *F* change with a mutant, whereas *F*_b_ does not and it can be measured by the force t/t generates. From Eq. 4, we get:
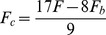
(5)

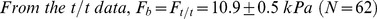
(6)The values for *F* is taken from averaged results of Figs. 2B and 3B at 5 mM MgATP and 8 mM Pi (standard activation).
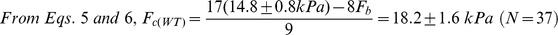


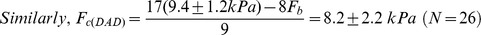
Thus, this analysis demonstrates that a cross-bridge with cMyBP-C generates (1.67±0.10) fold ( = *F*_c(WT)_/*F*_b_ = 18.2/10.9) force compared to that without cMyBP-C. It further demonstrates that a cross-bridge with DAD mutant generates (0.45±0.12) fold ( = *F*_c(DAD)_/*F*_c(WT)_ = 8.2/18.2) force compared to WT. Similar analysis demonstrates that a cross-bridge with ADA generates (0.86±0.15) fold force (*F* = 13.4±1.2 kPa, *F*_c_ = 15.6±2.2 kPa, N = 35), SAS generates (1.02±0.17) fold force (*F* = 14.9±1.3 kPa, *F*_c_ = 18.5±2.5 kPa, N = 27), and t/t generates (0.60±0.06) fold force (*F* = *F*_c_ = 10.9±0.5 kPa, N = 28) compared to WT.

Previous works have shown that the phosphorylation of cMyBP-C disrupts its interaction with myosin S2, and causes a reduction in the distance between S1 and actin. This reduction may increase the probability of cross-bridge formation, and may accelerate the force generation step (see review by [34]). Our study has demonstrated that phosphorylation of the two PKC sites (Ser273 and Ser302) strengthens the interaction between cMyBP-C and myosin S2, but diminishes its interaction with actin [35]. However, the differential effects of each phosphorylation site on binding to S2 are not completely understood. These molecular interactions between cMyBP-C, myosin S2, and actin must be the bases for cMyBP-C induced active force enhancement as we observed in WT *vs.* t/t. One likely explanation is an allosteric activation of myosin so that it interacts with actin in a more efficient way. This interaction can be modulated by phosphorylation of cMyBP-C by a variety of protein kinases. Therefore, it can be concluded that the purpose of cMyBP-C is to increase the active force, and cMyBP-C's phosphorylation is to modulate the amount of active force generation depending on which protein kinase is activated in cardiomyocytes at a particular moment.

The phosphorylation status of cMyBP-C or its mutants may interfere with phosphorylation of other myofilament proteins. Our results indicate that the decreased or absence of cMyBP-C phosphorylation is accompanied with a decreased RLC phosphorylation (in t/t, ADA and DAD) (Fig. 8), suggesting that the phosphorylation of the two proteins is inter-related. The proposed physical interaction between the M-domain of cMyBP-C and RLC [36] may contribute to the RLC phosphorylation triggered by cMyBP-C phosphorylation. The close correlation between cMyBP-C and RLC was also demonstrated in solution that cMyBP-C induced a significant increase in the actin-activated ATPase activity of both native and RLC-reconstituted myosin, but had no effect on RLC-deficient myosin [37]. The decreased RLC phosphorylation has no relation with the decreased tension per cross-bridge, because the latter only occurs in t/t and DAD, but not in ADA. However, we found that the rigor stiffness decreased in the t/t, ADA and DAD mice, all exhibited decreased RLC phosphorylation. Considering the influence of RLC phosphorylation on lever-arm stiffness [38], we conclude that the decreased RLC phosphorylation causes increased rigor stiffness, and this may be related to the altered distance of the myosin head to the thick filament [39]. A slightly decreased level of cMyBP-C phosphorylation was found in ADA [16], which is similar to our finding (Fig. 8).

A novel phosphorylation site Ser133 within the proline-alanine-rich linker domain between domains C0 and C1 was newly identified in human cMyBP-C [40]. This site could be phosphorylated by glycogen synthase kinase 3 beta (GSK3β), but not by protein kinase A (PKA). GSK3β has a modulating effect on sarcomere function by increasing the rate of tension redevelopment [40]. There is a possibility that additional phosphorylation sites in cMyBP-C may continue to be found, and its phosphorylation status may be associated with the phosphorylation of other sites.

The significantly decreased phosphorylation in cTnI, cTnT, RLC, and desmin, but not in cMyBP-C, was discovered in a study with frame shift mutation of cMyBP-C that was carried by hypertrophic cardiomyopathy patients [41]. In our mouse models, cMyBP-C protein was totally missing in the t/t mice and the mutated cMyBP-C was present at the similar levels in ADA, DAD, SAS, and WT mice (Fig. 7). This difference may cause a distinct effect on other myofilament proteins. An expression of skeletal TnT was found in the homozygous cardiac cMyBP-C-null (t/t) mice [42]. Specific antibody or mRNA probes are needed to identify these protein isoforms.

Several studies found that an increase in β-MHC expression in t/t mouse hearts [17], [43]–[45], which may affect observed rate constants [46]. Consequently, our observations may be confounded by a mutation and an isoform shift. For this reason, we studied the MHC isoform content in our previous work [17]. We found that our conclusions on ADA, DAD, and SAS were not significantly affected, because the isoform shift in them were small. In t/t, there was a significant increase in β-MHC expression. However, the involvement of isoform shift may not modify our conclusions on tension, stiffness, or magnitude parameters, because tension is not sensitive to the MHC isoform shifting [46]–[48], and stiffness and magnitude parameters are generally scaled with tension. The decreased force/cross-bridge (*T*_56_) in t/t is consistent with the observation that a partial (29±3%) extraction of cMyBP-C resulted in ∼30% reduction in maximal force [49].

The absence of cMyBP-C appear to cause a problem in cardiomyocyte organization, as demonstrated by disorganized and damaged sarcomere structures in t/t mice [13], which may cause further confounding problems. Consequently, the ultrastructural changes may partly account for the decreased force/cross-bridge in this report. These problems are difficult to solve in fiber studies on transgenic mouse hearts, and additional methods with future experimentations are expected to sort them out.

## Summary and Conclusions

DAD, similar to t/t, significantly reduces force per cross-bridge, apparent rate constant (2π*c*), and decelerates the cross-bridge detachment step. DAD mutant further demonstrates a reduced ADP release, and the elevated rate constant of the force generation step. These are different from t/t, which exhibited weakened ATP binding and enhanced Pi and ADP release. ADA and SAS mutants demonstrate insignificant impact on the force per cross-bridge, apparent rate constants, and the kinetics involved in steps 4–5. ADA slightly increases ATP binding and the cross-bridge detachment steps, and reduces ADP release, whereas SAS reduces ATP binding and promotes ADP release. Based on these findings, we conclude that the changes in the phosphorylation status of each site differentially affect the elementary steps of the cross-bridge cycle. In contrast to the limited influence on the ATP binding and the ADP release steps by ADA and SAS mutants, DAD produces broad effects by interfering with cross-bridge kinetics and negatively regulating force generation step, which largely mimic the t/t phenotype. Based on these findings, we conclude that it is the reduced force per cross-bridge, instead of the less numbers of strongly attached cross-bridges, that results in the reduced tension production in DAD and t/t mouse models. cMyBP-C, as an allosteric effector of myosin, increases the active force that cross-bridges generate, and its phosphorylation, which is regulated by variety of protein kinases, modulates the active force.
